# Mineral-associated organic matter is heterogeneous and structured by hydrophobic, charged, and polar interactions

**DOI:** 10.1073/pnas.2413216121

**Published:** 2024-11-08

**Authors:** Thomas R. Underwood, Ian C. Bourg, Kevin M. Rosso

**Affiliations:** ^a^Physical and Chemical Sciences Directorate, Pacific Northwest National Laboratory, Richland, WA 99352; ^b^Department of Civil and Environmental Engineering, Princeton University, Princeton, NJ 08544; ^c^High Meadows Environmental Institute, Princeton University, Princeton, NJ 08544

**Keywords:** soil carbon recalcitrance, soil organic matter, mineral-associated organic matter

## Abstract

The formation of mineral-associated organic matter (MAOM) is a key phenomenon that may explain the slow turnover rates of carbon in soil organic matter (SOM). Despite this, important details pertaining to the structure and dynamics of MAOM remain unknown. In the present study, we use replica-exchange molecular dynamics simulations to gain insight into the structure of MAOM on the surface of prototypical phyllosilicate clay and Fe-oxide minerals, montmorillonite and goethite, fine-grained minerals that strongly impact soil carbon dynamics in temperate and tropical regions, respectively. We examine the impact of aqueous chemistry through the presence of either Na^+^ or Ca^2+^ charge balancing counterions. Our results are consistent with the hypothesized multilayer sorption (“onion-skin”) model of MAOM and help to explain previous observations regarding the patchy distribution of SOM on mineral surfaces. In particular, the SOM coatings are partial and laterally heterogeneous, and water retains extensive access to mineral surfaces even when significant SOM sorption occurs. Low molecular weight neutral SOM molecules (<200 Da) infrequently interact with the mineral surfaces nor their sorbed organic matter coatings and are increasingly labile with decreasing molecular weight. This observation is inconsistent with a central feature of the predominant soil continuum model of SOM and suggests that further iterations of the conceptual model may be required.

## Significance

Soils contain the largest pool of organic carbon near the surface of the Earth and represent a natural carbon sink that helps to buffer against anthropogenic carbon emissions and land-use changes. Despite this, little is known regarding the molecular-level mechanisms of soil organic carbon recalcitrance. In the present study, we explore one of the key phenomena hypothesized to explain the stability of organic carbon in soils, namely mineral-associated organic matter. Using enhanced-sampling molecular dynamics simulations, we show that soil organic matter can be stabilized through a variety of mineral–organic interactions and through the intercession of organic–organic interactions.

Soils accommodate the largest pool of organic carbon near the surface of the Earth, conservatively estimated to be 1,500 PgC within the upper 100 cm horizon (1), and represent a natural carbon sink that helps buffer against anthropogenic carbon emissions and land-use changes (2). Carbon dating of soil organic matter (SOM) shows that certain forms of carbon are stored for many hundreds to thousands of years and are resistant to change; this material is often referred to as stable, recalcitrant, or “old” (3). The associated complex SOM turnover dynamics represent a key unknown in our ability to predict the future evolution of the soil carbon sink in a warmer, more extreme climate, and as a function of changing land management practices (4).

The historically predominant view of SOM has ascribed observations of slow carbon turnover dynamics (i.e., recalcitrance) to the formation of humic substances: large, polymeric, aromatic molecular structures that are resistant to enzymatic, bacterial, and fungal breakdown (5, 6). The last two decades, however, have seen a paradigm shift driven by evidence that humic-like substances constitute only a small fraction of the total amount of experimentally observed in situ SOM (7, 8). The emerging view, instead, is that SOM turnover dynamics are not an inherent material property associated with chemical recalcitrance (9) but rather, they emerge from a combination of greater ecosystem variables related to, but not limited to microbial ecology, enzyme kinetics, environmental conditions, and mineral/soil-matrix protection (10). An important milestone in this paradigm shift was the formulation of the soil continuum model (SCM) of Lehmann and Kleber (11). On this model, the gradual decomposition of large plant- and animal-derived residues results in the existence of a continuum of organic fragments (12, 13). As a consequence, SOM contains a high abundance of relatively small (<600 Da) organic molecules with comparatively high oxidation and solubility. These fragments should, in principle, be readily assimilated by soil biota, but they are hypothesized to persist due to their ability to form supramolecular aggregates that behave as large polymeric structures, and also, due to their tendency to associate with soil minerals, forming nominally stable structures known as mineral-associated organic matter (MAOM) (14–17).

The concept that SOM partitions to soil mineral surfaces to form MAOM is now considered to be one of the critical controls dictating soil carbon recalcitrance (18–21). For example, a meta-analysis of over 5,500 soil horizons revealed a global correlation between slow carbon turnover dynamics and the presence of fine-grained minerals, notably calcium-bearing phyllosilicate clay minerals in water-limited, alkaline, temperate soils (with soil pH ≳ 6.5) and nanocrystalline Fe- and Al-oxyhydroxides (hereafter referred to as metal-oxides) in water-rich, acidic, highly weathered soils (pH ≲ 6.5) (22). Furthermore, numerous experimental studies reveal the ability of SOM to partition into MAOM through two key mechanisms, one related to the coprecipitation of SOM with metal-oxides (23–25) and the other due to sorption of SOM to mineral surfaces (26–28). Yet, despite these remarkable observations, little is known in molecular detail about the mechanisms of MAOM formation, nor its ultimate structure, curtailing efforts to relate soil mineralogy to carbon storage capacity and limiting our ability to predict carbon turnover dynamics in large-scale climate models (29, 30).

**Fig. 1.**
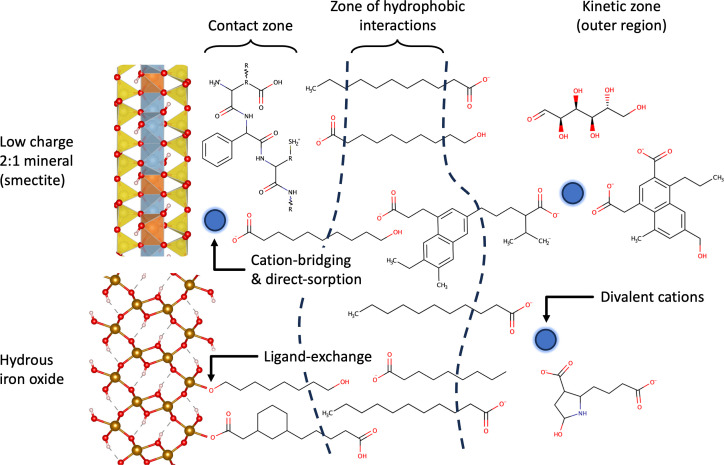
Schematic of the conceptual multilayer model proposed to stabilize SOM on the surfaces of 2:1 phyllosilicate clay minerals and Al-/Fe-oxyhydroxides. Recreated from Kleber et al. (32).

To the authors’ knowledge, the most detailed description of the structure of MAOM is that hypothesized by Sollins (31) and elaborated upon by Kleber (32). On this model, referred to here as the multilayer model (though also known as the zonal, onion-skin, or Kleber model), the persistence of SOM in fine-grained soils is ascribed to the formation of discrete layers about a mineral surface (Fig. 1). The first layer, named the “contact zone,” represents a region where amphiphilic SOM molecules are directly sorbed to the mineral surface, accumulating through a variety of bonding mechanisms. The sorbed amphiphilic molecules are hypothesized to coat the surface, presenting their hydrophobic tails outward, generating a second layer of SOM molecules sorbed to the mineral through entropically driven shielding of hydrophobic moieties, named the “zone of hydrophobic interactions.” Finally, a third layer or “kinetic zone” is hypothesized as the locus of any further auxiliary sorption of SOM to the mineral matrix through intermediate SOM–SOM interactions. The multilayer model formulates a coherent set of mutual interactions between SOM and soil minerals and has emerged as the predominant model of MAOM structure. However, as highlighted in a recent review (33), many of these observations may also be explained with simpler structural descriptions of MAOM, such as monomer adsorption; monolayer adsorption; and multilayer adsorption (34). Consequently, numerous questions remain regarding the structure of MAOM. What is the nature of sorbed SOM to mineral surfaces, does it sorb as monomers, as discrete films, or as a more complex heterogeneous phase? Why does SOM sporadically appear to form partial and patchy distributions on mineral surfaces (35, 36)? How do aqueous chemistry and SOM composition impact the formation of MAOM at the scale shown in Fig. 1? Notably, aqueous calcium ions and nitrogen-bearing functional groups in SOM have been empirically observed to enhance the retention of organic matter in the form of MAOM (36–38). These questions form the basis of the present study, in which we aim to examine the structure of MAOM using computational chemistry, and in particular, classical molecular dynamics (MD) simulations of a complex SOM model carefully equilibrated using enhanced sampling techniques.

Molecular dynamics simulations have been extensively used to gain insight into the structure of complex organic systems including the self-assembly of bioinspired supramolecular aggregates (39), the formation of lipid bilayers (40), and the aggregation of extracellular polymeric substances (41). Applications of this technique to help resolve the structure of SOM coatings on mineral surfaces remain minimal, however, for two important reasons. First, a well-defined molecular structure of SOM needs to be clarified as an input to the simulations. Unfortunately, common SOM chemical characterization techniques such as infrared spectroscopy and NMR reveal only the average abundance of different chemical functional groups (42). More recently developed techniques, such as Fourier-transform ion cyclotron resonance mass spectrometry, can provide stoichiometries of each molecule in a soil sample but cannot reveal the structure nor precise relative abundance of each molecule (43). Despite this, several attempts have been made to produce a molecular structure of SOM applicable for MD simulations albeit most of these efforts have focused on recreating the structure of SOM within the historic framework of humic substances (44–50). Notable among these are the Temple-Northeastern-Birmingham model and the Vienna Soil Organic Matter Model (VSOMM and VSOMM2) (51–53). Second, although MD simulations probe appropriate length scales to examine the validity of the multilayer model (i.e., on the order of several nanometers), they are limited by computational cost to timescales of hundreds of nanoseconds. The slow dynamics of mineral-associated organic matter suggest relaxation times on the order of hours to years (28). Previous MD simulation studies of MAOM have been limited to sampling of so-called brute force production runs, which are susceptible to generating ensembles and structures pinned in metastable states (54–56). Fortunately, studies of other slow processes, such as protein folding or the formation of biomolecular condensates, have led to the emergence of enhanced sampling MD simulation approaches that can accelerate the generation and sampling of well-equilibrated ensembles representative of these slow processes (57). A prominent method among these enhanced sampling techniques is the replica-exchange MD (REMD) simulation method (58).

In the present study, we elucidate the structure of MAOM at environmentally relevant temperatures, from 300 K to 335 K in 5 K increments, using REMD enhanced molecular simulations. Using this enhanced sampling technique, we examine SOM–mineral interactions. The SOM model examined is derived from the molecular structure of dissolved organic matter developed by Devarajan (59), hereafter referred to as the Oak Ridge National Laboratory (ORNL) model, notable for its complexity and compatibility with the soil continuum model of Lehmann and Kleber (11). Each unique model contains an equal amount of SOM—consistent with the work of Devarajan (59), and while previous research has shown that the partitioning and clustering of hydrophobic moieties can be related to a minimization in the solvent-accessible apolar surface area of the hydrophobic phase (60), the effects of absolute abundance and relative abundance of each SOM molecule has been reserved for future work. In particular, we examine the structure of SOM on the basal surfaces of montmorillonite, a prototypical example of the 2:1 layer type phyllosilicate clay minerals that strongly impacts SOM dynamics in temperate soils, as well as the energetically stable (010) surface facet of the Fe-oxyhydroxide mineral goethite, an analog of the nanocrystalline Fe-oxides that correlate with SOM dynamics in heavily weathered environments (22). We note that, even with appropriate atomistic models and enhanced sampling techniques, we can expect certain limits and challenges. First, classical MD simulations are unable to predict changes in covalent bonding. Subsequently, we are limited to examine the properties of MAOM related to van der Waals, Coulomb, and hydrophobic interactions, and are unable to examine phenomena related to mineral precipitation and dissolution. To incorporate changes in covalent bonds one would need to use more computationally expensive techniques such as reactive molecular dynamics or density functional theory, methods which are currently infrequently used to examine the properties of SOM and MAOM (61). Second, while enhanced sampling techniques such as REMD can generate equilibrium structures, and by using standard MD simulations, we can examine the short-term dynamics of these structures on the time-scale of 100 s of nanoseconds, we cannot model the expected slow rearrangement dynamics within SOM aggregates or coatings on time scales of days. Examinations of the molecular-level rearrangements of MAOM on time-scales greater than microseconds would require an upscaled description of the pertinent systems using techniques such as coarse-grained MD simulations (62). Such coarse-grained approaches have not yet been developed for systems that contain both SOM and soil minerals. The all-atom simulation results presented here aim to provide detailed fundamental insight into the structure of MAOM while also providing a benchmark for the development of future coarse-grained models.

## Results

### The Structure of MAOM Coatings.

**Fig. 2.**
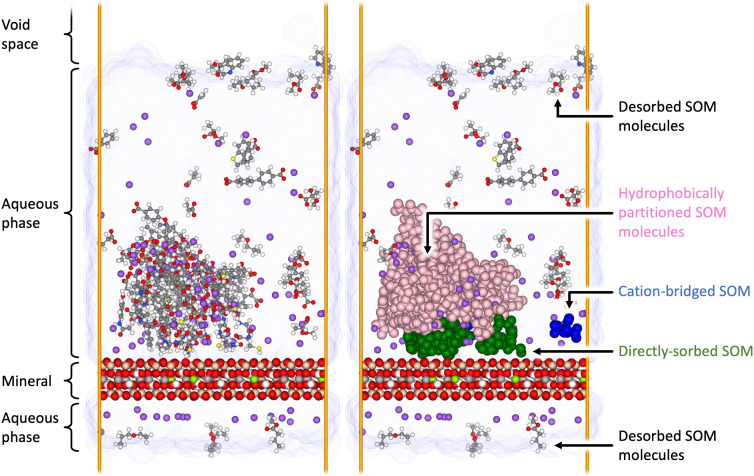
A representative configuration of the ORNL SOM model interacting with a sodium charge-balanced montmorillonite surface at 300 K (*Left*). Small spheres represent SOM atoms (C, O, H, N, S, and P in gray, red, white, blue, yellow, and orange, respectively); large spheres represent the clay mineral atoms (O, H, Si, Al, Mg in red, white, tan, taupe, and light green, respectively); purple spheres are charge-compensating Na^+^ ions; water molecules are not presented for clarity, however the extent of the water phase is presented in the transparent blue region. The second panel (*Right*) illustrates our capacity to calculate and identify the sorption and clustering of SOM monomers through cation bridging (blue), SOM in direct contact with the surface (dark green), and SOM clusters sorbed through auxiliary SOM molecules (pink).

Fig. 2 presents a configuration of the ORNL SOM model interacting with a Na-montmorillonite surface at 300 K, from which we observe several high-order details that are consistent across all the systems and temperatures examined. First, we observe the self-assembled aggregation of SOM molecules into a large primary cluster. Second, numerous SOM molecules do not appear to aggregate within this cluster. Third, we observe an affinity between a small fraction of the SOM molecules and the liquid–vapor interface. We propose that this may hold important implications for the fate and transport of SOM in soils with cyclical patterns of saturation, i.e., recurring patterns of soil wetting and desiccation may promote the removal of certain forms of SOM from mineral surfaces. This observation is consistent with the oft-overlooked concept of a microbial volatilome (63) and is consistent with observations that adsorption at the air–water interface can dictate the transport of organic contaminants in the vadose zone (64). Fourth, multiple SOM molecules have evaporated from the liquid phase, have passed through the vapor phase of the system, through the periodic boundaries of the model, and have settled on the opposing side of the mineral surface. Analysis shows that the evaporated molecules are always neutral and consequently do not generate any artificial dipole moment across the simulation cell, which could negatively impact our results. Finally, in all of the systems examined, we observe prevalent interactions between the mineral surface and the SOM phase. Although SOM molecules can be observed infrequently sorbed as monomers (as illustrated by the sole cation-bridged blue-tagged molecule in Fig. 2), they predominantly form hydrophobic clusters (pink-tagged molecules in Fig. 2) that attach to mineral surfaces through the intercession of a few SOM molecules bound through a variety of interaction mechanisms (as illustrated by the green-tagged molecules in Fig. 2). In the following section, we discuss the particular molecular details pertaining to SOM self-aggregation, followed by the nature of the contact zone and the consequent SOM–mineral interactions particular to each system. Additionally, we observe no significant qualitative difference between the interactions of SOM and each mineral as a function of temperature. Consequently, all proceeding results and discussions have been calculated as an average over all the temperature configurations examined for each mineral and aqueous chemistry ensemble.

### The Hydrophobic Cluster.

**Fig. 3.**
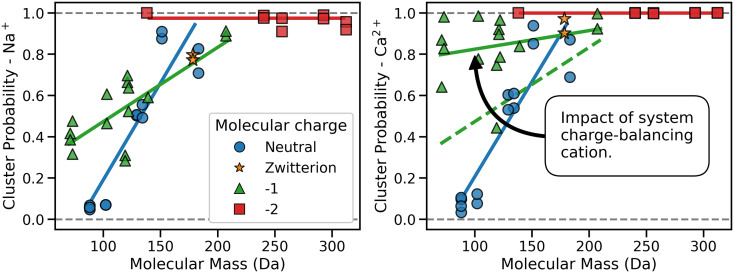
The probability of each SOM molecule partitioning into the largest cluster of the system as a function of molecular mass and aqueous chemistry. Results are presented for sodium charge-balanced systems (*Left*) and calcium charge-balanced systems (*Right*). Each data point is averaged over both montmorillonite and goethite models and over all temperatures. Lines of best fit are presented to guide the eye. The introduction of calcium ions increases the likelihood of smaller charged molecules being incorporated into the largest primary cluster through the formation of a kinetic zone (green lines in the *Right* panel).

In all the systems examined we observe the aggregation of a large primary cluster of SOM molecules. Approximately 61% of the SOM molecules in the sodium charge-balanced systems are contained within the largest cluster, increasing to 75% of SOM molecules in the calcium charge-balanced systems. SOM molecules with a mass greater than 200 Da are almost always observed as constituents in the primary cluster. Beneath this cutoff, we observe a linear correlation between the molecular mass of an SOM molecule and its probability of existing in the primary cluster in the sodium charge-balanced systems as shown in the *Left* panel of Fig. 3. Notably, this result is in direct contrast to the SCM of Lehmann and Kleber (11), which proposes an inverse relationship between the mass of SOM molecules and their partitioning into hydrophobic supramolecular aggregates. Our results suggest this relationship, if it exists, reverses at molecular weights below 200 Da, an observation that corroborates multiple batch sorption experiments on the molecular-weight fractionation of dissolved organic matter about metal-oxide and clay mineral surfaces (65, 66).

The monotonic increase in cluster probability with constituent SOM molecular mass suggests our data may be analyzed utilizing Spearman correlation coefficients. Consequently, we have extended our analyses to compare the clustering probability of each SOM molecule against multiple key chemical features characterized using the cheminformatics suite RDKit, octanol–water partition coefficients calculated using XLOGP3 (67), and using locally developed tools to calculate a molecule’s polar and apolar solvent-accessible surface area (SASA). Spearman correlation coefficients comparing an SOM molecule’s clustering probability against each of these unique properties are presented in *SI Appendix*. Henceforth, references to correlations in the remainder of the manuscript are directly related to the magnitude of these Spearman correlation coefficients.

Our cheminformatics correlation analyses reveal a strong correlation between the likelihood of clustering and a molecule’s degrees of unsaturation (viz. its double bond equivalents), as well as the number of aromatic rings contained within the molecule. Additionally, we observe correlations between a molecule’s predicted octanol–water partition coefficient, as well as its apolar surface area, and the probability of it clustering out of solution. Consequently, we conclude that the primary mechanism for the clustering of SOM is through hydrophobic partitioning, whereby the SOM molecules are driven to aggregation so as to minimize the interactions between their hydrophobic moieties and the bulk solvent phase.

While the composition of the primary cluster in the calcium charge-balanced systems is near-identical to that of the sodium charge-balanced systems, there is an increased likelihood that small negatively charged monovalent SOM molecules can incorporate into the cluster in the presence of aqueous calcium, as shown in the *Right* panel of Fig. 3. Such an increase is concordant with the multilayer model’s hypothesis of the formation of a kinetic zone and is in agreement with previous experimental and MD simulation observations of organic matter aggregation in the presence of calcium ions (68–70). Cheminformatic correlation analyses for the calcium charge-balanced systems present similar trends as the sodium systems. However, we additionally observe a strong correlation between the negative charge of a molecule, as well as its number of carboxylic acid functional groups, and the probability of a molecule being in the primary cluster. Consequently, we propose that the addition of calcium into a system does not perturb the preexisting hydrophobic nature of SOM self-assembled clusters, rather, it additionally appends SOM upon the preexisting hydrophobic cluster through the formation of divalent cation bridges.

### The Contact Zone.

**Fig. 4.**
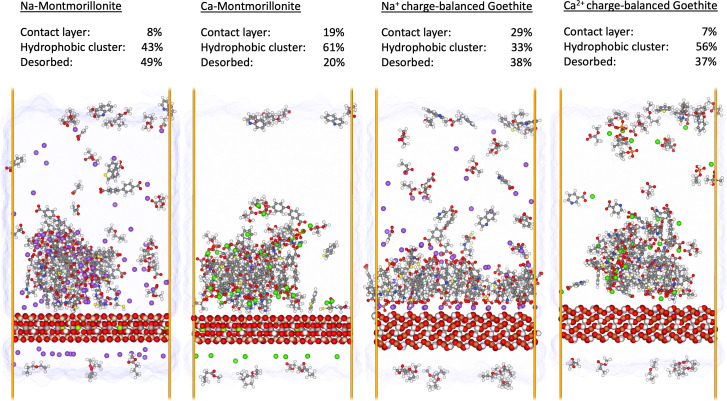
Representative snapshots of the ORNL SOM model interacting with different mineral surfaces. Colors are identical to Fig. 2, with the addition of aqueous Ca^2+^ (purple) and mineral Fe (orange). From *Left* to *Right*: Na-montmorillonite, Ca-montmorillonite, goethite interacting with Na^+^ charge-balanced SOM, and goethite interacting with Ca^2+^ charge-balanced SOM. Above each snapshot is the average percentage of adsorbed and desorbed OM averaged across all simulated configurations and temperatures.

Fig. 4 presents the sorption of SOM to Na-montmorillonite; Ca-montmorillonite; Na^+^ charge-balanced goethite; and Ca^2+^ charge-balanced goethite. For clarity, we note that the montmorillonite surfaces contain a net permanent negative charge due to isomorphic substitutions of octahedral aluminum atoms for magnesium atoms, while the goethite mineral is net neutral. Additionally, the ORNL SOM model has a net negative charge. In the montmorillonite systems, aqueous cations charge-balance both the mineral and the SOM phase, while in the goethite systems, aqueous cations charge-balance the SOM phase solely. In all systems, we observe the formation of a primary SOM phase which subsequently sorbs to the mineral surface. Monomer sorption is also observed for all models, albeit such events occur infrequently and are short-lived. The average amount of SOM sorbed to each mineral surface, either directly or through auxiliary SOM–SOM interactions, has been calculated and is also presented in Fig. 4. Additionally, we observe the formation of a kinetic zone due to the formation of divalent cation bridges between negatively charged functional groups in the SOM, though these values are incorporated into the “hydrophobic cluster” percentages presented in Fig. 4.

To further analyze these systems we have compared the Spearman correlation coefficients between important chemical features of every SOM molecule against the likelihood that the SOM molecule is *bridging* between the mineral and an auxiliary, hydrophobically partitioned, SOM molecule (c.f. molecules that are tagged as green in Fig. 2).

The strongest indicator of sorption and bridging to the surface of Na-montmorillonite is the presence of nitrogen in an SOM molecule, primarily in the form of positively charged amine groups (prevalent among amino acids and proteinaceous material) as well as nitrogen-containing aromatic rings such as pyridines. We additionally see a moderate positive correlation between both the overall degree of unsaturation (double bond equivalents) and aromaticity and the prevalence of bridging events. Finally, we observe a small correlation between the positive charge of an SOM molecule and the prevalence for bridging events. Since there are no positively charged SOM molecules in our simulations (positively charged primary-amine functional groups are contained within zwitterions in the ORNL SOM model), this result indicates instead, a repulsion of negatively charged SOM molecules from the bridging phase, consistent with previous MD simulation observations of carboxylic acids interacting with Na-montmorillonite (71). The picture painted here is one of direct sorption between nitrogen-containing (positively charged or positively polar) functional groups and negatively charged smectite surfaces supplemented by hydrophobic interactions between unsaturated and aromatic moieties with the hydrophobic patches of the mineral surface, results that are consistent with literature observations of the sorption of organic contaminants to smectite minerals (72, 73). Furthermore, the Spearman correlation coefficients observed for Na-montmorillonite bridging events are notably smaller compared to those observed for the subsequently discussed surfaces, suggesting that such bridging events are less stable compared to SOM interactions with Ca-montmorillonite and goethite. We subsequently postulate that while SOM can be stabilized by the surface of Na-montmorillonite, this stabilization is likely weaker than in the other systems considered in the present study.

The abundance of SOM molecules in the contact zone of montmorillonite is more than doubled in the presence of calcium. In direct contrast to the Na-montmorillonite system, for Ca-montmorillonite we observe a strong correlation between the negative charge of an SOM molecule and its occurrence in the bridging phase. In particular, we observe a correlation between both the quantity of carboxylic acids and highly polar carbonyl oxygens and the prevalence of SOM bridging events. This occurs at the expense of the nitrogen-containing functional groups and aromatic groups prevalent in the bridging phase of the Na-montmorillonite system. The result indicates the importance of divalent cation bridges between SOM and the negatively charged mineral surface, in a similar manner to how calcium can stabilize the bulk SOM phase as described above, and is consistent with observations that Ca-montmorillonite can sorb petroleum compounds rich in carboxylic acid (71). Further examination of the correlation coefficients suggests that the divalent cation bridges observed in the Ca-montmorillonite system are stronger than, and tend to override, rather than supplement, the direct ionic and hydrophobic interactions observed in the Na-montmorillonite system.

**Fig. 5.**
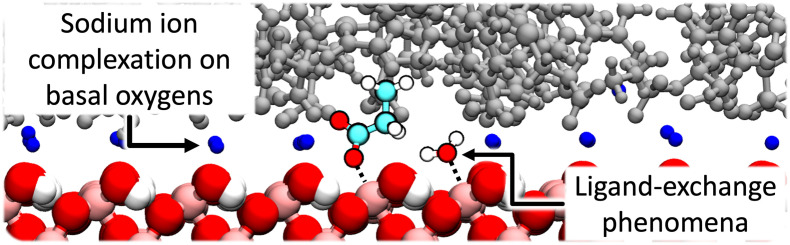
A close-up configuration of the Na^+^ charge-balanced goethite system interacting with the ORNL SOM model. Sodium ions can be observed directly sorbed upon the basal oxygen atoms of the goethite as inner-sphere surface complexes. Also highlighted is an illustrated example of ligand exchange, whereby structural Fe replaces one of its OH_2_ ligands with an oxygen atom from the SOM phase. Other water molecules and surface OH_2_ ligands are not presented for clarity.

The key indicators for sorption and bridging to goethite, regardless of counterion type, are similar to that of Ca-montmorillonite, namely SOM negative charge; the presence of carboxylic acids; and the presence of polar carbonyl oxygens. In fact, we observe the strongest correlation of these coefficients among all observed systems in the scenario of Na^+^ charge-balanced SOM on goethite. The result is surprising, considering our model of goethite is neutral and contains no permanent dipole moment; however, the phenomena may be explained upon examination of the complexation of sodium ions about the goethite surface in Figs. 4 and 5. Notably, we observe strong inner-sphere sorption of sodium ions to the surface of goethite, a phenomenon caused by the strong templating of water molecules about the (010) surface of goethite as highlighted by Criscenti (74). The sorption and bridging of SOM then follows from ionic interactions between the sorbed sodium ions and the negatively charged and polar moieties of SOM molecules. As a consequence of the strong binding between the (010) surface of goethite and sodium ions, we observe significant quantities of SOM directly sorbed to the goethite mineral in the Na^+^ charge-balanced system: almost twice as much as in the Ca-montmorillonite case and four times as much as in the Na-montmorillonite and Ca^2+^ charge-balanced goethite system (upper text in Fig. 4). Despite this, the absolute amount of SOM sorbed to the Na^+^ charge-balanced goethite (i.e., that which is directly sorbed and that which is sorbed through intermediate SOM–SOM interactions) is smaller than in the Ca^2+^-SOM charge-balanced goethite or montmorillonite systems, confirming that whereas Na^+^ can effectively bridge between the goethite surface and anionic SOM molecules, Ca^2+^ more effectively bridges between anionic SOM moieties. This is clear from the snapshot of the sodium charge-balanced goethite system in Fig. 4, whereby carboxylic acid functional groups arrange as to (a) complex with the sodium ions sorbed to the surface, and (b) to point outward toward the external aqueous phase.

Finally, we observe numerous occurrences of ligand-exchange phenomena between the SOM phase and the goethite surface, Fig. 5. In such instances, the iron of the goethite mineral, which is typically sixfold coordinated with five mineral oxygen atoms and one OH_2_ ligand (topologically identical to a water molecule in the presented MD simulations), substitutes its coordination from the OH_2_ oxygen atom with an oxygen atom from a carboxylic acid group of the SOM phase. Such reactions are expected based on density functional theory simulations and spectroscopic data (75, 76), but to the authors’ knowledge, this specific ligand-exchange event has not previously been observed using classical MD simulations. We note that while the semiempirical interatomic potential models used here have been extensively validated for mineral–water and water–organic interactions, the combination of forcefields in terms of its ability to predict the detailed features of mineral–water–organic interactions has been less well examined. Our observation of ligand-exchange reactions provides confidence in our combination of force-fields, yet we caution that more quantitative validations would be required to establish whether our simulation approach accurately predicts the rates and energetics of such ligand-exchange reactions.

Compared to the sodium charge-balanced goethite system, we observe similar (albeit slightly smaller) correlations between the presence of negatively charged functional groups and the sorption and bridging of SOM for Ca^2+^ charge-balanced goethite. Notably, however, this sorption is not due to the complexation of SOM with cations sorbed to the surface, as shown by the lack of sorbed calcium ions to the goethite surface in Fig. 4. To analyze this further we have quantified the total surface area coverage of water, SOM, and ions about the *xy*-plane in the first four discrete atomic layers above each mineral surface (data presented in *SI Appendix*). For Ca^2+^ charge-balanced goethite, we observe that a significant majority of the planar surface area in the first four discrete atomic layers above the surface is dedicated to water. In fact, only a small fraction, less than 1%, of the total surface area is attributed to calcium ions in any of the first four discrete atomic layers. Consequently we hypothesize that the key mechanism driving SOM sorption and bridging to the Ca^2+^ charge-balanced goethite system is through water bridging and is a consequence of the (010) surface of goethite forming large quantities of strong hydrogen bonds with the surrounding solvent phase in the absence of sodium ions (74). In contrast, we observe a significant amount of surface area attributed to sodium ions in the planes above the goethite surface for the sodium charge-balanced system. Finally, we observe that, while each mineral phase sorbs a significant quantity of SOM, the minerals all retain a large quantity of water adjacent to the surface, greater than 80% in the first atomic layer adjacent to the mineral for all systems examined. These surface area calculations, along with the simulation visualizations, suggest deep level of complexity regarding the structure of MAOM. First, that the SOM coatings only partially cover the mineral surface and are laterally heterogeneous, a result that concords with the patchy distribution of SOM on hydrated minerals observed with transmission electron microscopy (35, 36). Second, that even while significant SOM sorption occurs on all the examined mineral surfaces, the minerals remain overwhelmingly hydrated and water retains extensive access to the surface.

## Implications

The assemblage of organic matter on mineral surfaces is now considered to be one of the most important facets dictating slow carbon turnover rates and recalcitrance in soils. Recent literature highlights the need to understand the structure of this mineral-associated organic matter, since the Occam’s razor explanation, monomer sorption, is frequently sufficient to describe experimental observations (33). In this study, we present a picture of mineral-associated organic matter that is extremely heterogeneous, eschewing the hypothesis that SOM sorbs to minerals as monomers, as monolayers, or as uniform films.

Our observations appear to directly confirm the essential chemical tenets of the multilayer adsorption model proposed by Kleber (32), namely that amphiphilic soil organic matter molecules bind to mineral surfaces, presenting their hydrophobic tails to solution. Additional SOM is subsequently sorbed to the mineral surface via intermediate hydrophobic partitioning and through the formation of a kinetic zone in the presence of calcium ions. Furthermore, our results introduce a previously unseen level of additional complexity beyond the original formulation of the multilayer model, namely, that SOM coatings on mineral surfaces are both partial and laterally heterogeneous, and are capable of interacting with minerals through a wide variety of bonding mechanisms. These conclusions help explain the observations of nonuniform and patchy distributions of SOM on mineral surfaces (35, 36). Additionally, we highlight that significant sorption of SOM can occur on the surfaces of topical soil minerals even while the mineral surfaces remain overwhelmingly hydrated.

Our results further highlight the key organo–mineral interactions dictating the stability of MAOM as a function of both mineral type and aqueous chemistry. Nitrogen-rich moieties play a particularly important role in the binding of SOM to Na-montmorillonite. This result lends support to the observation that elevated levels of nitrogen-rich SOM moieties have been observed in the contact zone of MAOM (36). We note, however, that this effect is overridden by the formation of divalent cation bridges between SOM and the smectitic mineral in the presence of aqueous calcium ions. Additionally, we observe a large quantity of SOM sorbed to the (010) surface of goethite through cation bridging of sodium ions, a result that is unintuitive, yet is consistent with previous observations of strong inner-sphere binding of sodium ions to goethite (74).

Aqueous chemistry plays an extremely important role in the formation and stability of MAOM, both dictating the key bonding mechanisms between SOM and minerals, as well as enhancing the amount of sorbed SOM through the formation of a kinetic zone. The increase in stability and storage of SOM in the presence of aqueous calcium ions agrees with previous experimental observations (37, 38). Our analyses reveal that aqueous calcium ions act as to append additional SOM to a preexisting, hydrophobically partitioned, cluster of SOM. The effect is particularly pertinent for light-weight monovalent anionic SOM molecules, which are able to effectively dress the hydrophobic cluster in the presence of calcium ions, but not in the presence of sodium ions.

Additionally, we observe an affinity between certain SOM molecules and the liquid–vapor interface. While a detailed examination of this process is beyond the scope of the current work, we highlight this observation as it may hold important implications for the fate and transport of SOM and MAOM in environments where soils are subject to patterns of cyclical wetting and desiccation.

Finally, we highlight an inconsistency between our observations and the soil continuum model proposed by of Lehmann and Kleber (11). In the conceptual model, fragments of soil organic matter are continuously broken down into ever smaller constituents. The model proposes an inverse correlation between the molecular weight of a molecule and its partitioning into supramolecular clusters and/or MAOM, thus introducing a mechanism by which these lightweight SOM constituents can be protected from further metabolic breakdown. Yet, beneath a threshold at approximately 200 Da, we observe a positive-linear correlation between the molecular mass of a constituent SOM molecule and its likelihood of partitioning into a hydrophobic cluster and hence sorbing to a mineral surface. Our results suggest that a critical reanalysis of the soil continuum model may be required, with particular attention paid toward the behavior of light-weight, readily assimilable, biopolymers and monomers.

In conclusion, we have applied replica-exchange molecular dynamics simulations to gain insight into the structure of MAOM on the surface of prototypical phyllosilicate clay and Fe-oxide minerals as a function of aqueous chemistry. In doing so, we have successfully detailed and expanded upon numerous experimental observations regarding the structure of MAOM. Despite our successes, we would like to reiterate that certain challenges remain related to our current methodology and discuss how future developments could help alleviate such limitations. First, classical MD simulations are unable to predict changes in covalent bonding and subsequently cannot be expected to model reactive environments. To incorporate changes in bonding environments one would need to use more computationally expensive techniques such as reactive molecular dynamics or density functional theory, methods which are currently limited in both time- and length-scale, and are consequently infrequently used to examine the properties of SOM and MAOM (61). We posit that future work incorporating reactive environments including both SOM and aqueous inorganic material would offer a large boon in understanding the phenomenon of SOM recalcitrance through the coprecipitation of organic matter with short-range ordered Fe- and Al-oxides in moderately acid soils (22). Second, while enhanced sampling techniques such as the replica-exchange algorithm used in the present study can generate equilibrium structures, and by using standard MD simulations, we can examine the short-term dynamics of these structures on the time-scale of 100 s of nanoseconds, we cannot yet model the expected slow rearrangement dynamics within SOM aggregates or coatings on time scales of days. Examinations of the molecular-level rearrangements of MAOM on time-scales greater than microseconds would require an upscaled description of the pertinent systems using techniques such as coarse-grained MD simulations (62). Coarse-grained approaches have not yet been developed for systems that contain both SOM and soil minerals but would offer a path forward for exploring longer time scale processes that could contribute to the phenomenon of SOM recalcitrance, such as occlusion of organic matter in nanoporous fine-grained systems.

## Materials and Methods

**Fig. 6.**
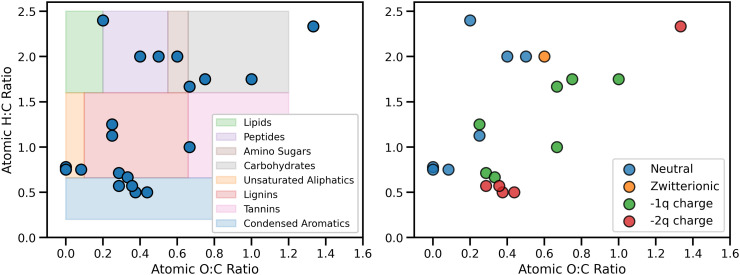
van-Krevelen diagrams of the SOM model considered in this study categorized by SOM molecule type as per Devarajan et al. (59) (*Left*) and by charge (*Right*).

The SOM model examined in the present study is derived from the molecular structure of dissolved organic matter developed by Devarajan (59). This model is notable for its compatibility with the soil continuum model of Lehmann and Kleber (11), in the sense that it contains a mixture of organic molecules with molecular weights ranging from ≈70 Da to 300 Da. Our model includes 20 unique molecules presented in the form of a van Krevelen diagram in Fig. 6. Additionally, the molecular structure of each molecule is presented in *SI Appendix*. Each simulation contains 84 SOM molecules in total, 4 duplicates of each SOM molecule, and 8 duplicates of the peptide glycylcysteine, multiplied to exemplify the particular role that nitrogen-containing functional groups may have on the structure of MAOM.

Four separate systems (two different minerals, each simulated with either Na^+^ or Ca^2+^ charge-balancing ions) were examined using eight unique replicas of each system. The SOM model was simulated in an aqueous phase interacting with the standard charge neutral 1×1 termination of the (010) surface of the Fe-oxyhydroxide mineral goethite, with protonation states imposed to mimic a pH of 5.5, or with the basal surface of the 2:1 layer-type phyllosilicate mineral montmorillonite, setup to mimic a pH of 7.5. In addition, the protonation of each SOM molecule was adjusted to its most prevalent state for each pH. These systems, at their concomitant pH levels, approximate the key variables hypothesized to minimize SOM turnover rates in highly weathered (e.g. tropical or subtropical) soils with pH ≲ 6.5 vs. moderately weathered soils with pH ≳ 6.5 (22). We note that the *Pbnm* (010) surface of goethite examined in this study is frequently presented as the (100) surface in the *Pnma* space group in other works (76). A vapor phase was placed above the aqueous–SOM phase to mimic unsaturated conditions and to minimize interactions between the periodic images of the simulated systems. Full details regarding the initial setup of these systems can be found in *SI Appendix*.

Molecular dynamics simulations were performed using GROMACS 2022.4 on the Department of Energy’s (DOE) Deception High Performance Compute cluster at the Pacific Northwest National Laboratory, utilizing the CHARMM3.6 derived CGenFF force field for SOM molecules, the ClayFF force field for minerals and aqueous cations, and the SPC/E model for water. All simulations were carried out in the canonical ensemble with a time step of 1 fs using periodic boundary conditions, with electrostatic and Lennard-Jones interactions truncated at a cutoff distance of 1 nm, and with long-range interactions extrapolated using the particle–mesh Ewald summation method. Interaction potentials were shifted at the cutoff to avoid any nonlinear jumps in forces between atoms. Each model was initialized subject to an energy minimization followed by a brief (100 ps) equilibration in the canonical ensemble. Subsequently, the replica exchange procedure was utilized among the eight unique replicas of each system to aid in finding representative configurations of MAOM. A 100 ns REMD simulation was performed with Monte Carlo configuration swaps attempted every 1 ps. Temperatures of the eight replicas of each system ranged from 300 K to 335 K in 5 K increments. Finally, each 5 K incremental simulation was continued using the traditional MD technique for 200 ns, with configurations saved every 5 ps for data analysis.

To analyze the resulting configurations, we have developed a clustering algorithm capable of describing whether an SOM molecule is aggregated on a mineral surface and, if so, whether it is directly sorbed, bridged through an intermediate cation, or sorbed through auxiliary intermediate SOM molecules. Details pertaining to the sorption and clustering of SOM have been correlated to the chemical features of each SOM molecule calculated using the RDKit cheminformatics toolkit. In-house tools were utilized to calculate the SASA of each molecule, discretized into polar and apolar SASAs. The polar SASA of a molecule was defined as that corresponding to oxygen or nitrogen atoms and the apolar surface area is that corresponding to other atoms. Hydrogen atoms were excluded in this calculation. The octanol–water partition coefficient of each SOM molecule was predicted using the XLOGP3 software package (67). Additionally, we have developed a method utilizing Voronoi tessellation to discretize the surface area coverage of each mineral. In so doing, we quantify how much of a mineral’s surface area is interfaced with water, with ions, and with SOM molecules. These surface area calculations were performed for the first four atomic layers above each mineral surface using the PyTIM algorithm to detect interfacial atoms (77). Full details of all algorithms are described in *SI Appendix*.

## Supplementary Material

Appendix 01 (PDF)

## Data Availability

In-house code used to determine the clustering of SOM upon mineral surfaces has been deposited on Github (78). All other data are included in the manuscript and/or supporting information.
