# Supplementary material for: Mineral-associated organic matter is heterogeneous and structured by hydrophobic, charged, and polar interactions
Source: Proc Natl Acad Sci U S A. 2024 Nov 8;121(46):e2413216121. doi: 10.1073/pnas.2413216121 (PMC11573572; doi:10.1073/pnas.2413216121)
Supplement: Supplementary file 1 — Appendix 01 (PDF) [file pnas.2413216121.sapp.pdf]

Supporting Information for

Mineral-Associated Organic Matter is Heterogeneous and Structured by Hydrophobic, Charged, and Polar Interactions

Thomas R. Underwood, Ian C. Bourg, Kevin M. Rosso

Thomas R. Underwood.
E-mail: thomas.underwood@pnnl.gov

This PDF file includes:

Supporting text
Figs. S1 to S8
Tables S1 to S2
SI References

Supporting Information Text

Simulation Details

The Soil Organic Matter (SOM) Model. The SOM model examined in the present study is derived from the model structures DOM1 and DOM2 of dissolved organic matter developed by Devarajan *et al.*, (1), hereafter referred to as the Oak Ridge National Laboratory (ORNL) model. The model contains 20 unique molecules presented in the form of a van Krevelen diagram in main article and in Table S1 below. Additionally, the molecular structure of each molecule is presented in Figure S1.

Table S1. The composition of the SOM model used in the present study. In all instances, every simulation contains nMol replicates of each SOM molecule.

Molecule	Charge (*e*)	Mass (Da)	C	H	O	N	S	P	nMol
Benzoate	-1	121.1	7	5	2				4
Pyridine-3-carboxylate	-1	122.1	6	4	2	1			4
2-hydroxy-2-methoxypropanoate	-1	119.1	4	7	4				4
2-methoxypropanoate	-1	103.1	4	7	3				4
[1,1'-biphenyl]-4,4'-dicarboxylate	-2	240.2	14	8	4				4
2-[4'-(carboxylatocarbonyl)-[1,1'-biphenyl]-4-yl]-2-oxoacetate	-2	292.6	16	8	6				4
4-(4-carboxylatophenoxy)benzoate	-2	312.2	16	8	7				4
2-{4-[4-(carboxylatocarbonyl)phenoxy]phenyl}-2-oxoacetate	-2	256.2	14	8	5				4
1-ethoxypropane	0	88.2	5	12	1				4
3-[4-hydroxy-3-(propan-2-yl)phenyl]propanoate	-1	207.2	12	15	3				4
10H-phenoxazine	0	183.2	12	9	1	1			4
Glycylcysteine	0	178.2	5	10	3	2	1		8
Propan-2-yl phosphate	-1	139.1	3	8	4			1	4
Propan-2-yl phosphate	-2	138.1	3	7	4			1	4
Methyl butanoate	0	102.1	5	10	2				4
Methyl propanoate	0	88.1	4	8	2				4
N-(4-hydroxyphenyl)acetamide	0	151.2	8	9	2	1			4
Prop-2-enoate	-1	71.1	3	3	2				4
Propanoate	-1	73.1	3	5	2				4
Quinoline	0	129.2	9	7	1				4
1-benzothiophene	0	134.2	8	6			1		4

We note that the peptide *glycylcysteine* has been modeled as a monomer in the present study. This is in contrast to the ORNL model which examines glycylcysteine as peptide bonded dimers. As a consequence, we have utilized 8 replicas of glycylcysteine in each model in our study (see Table S1), compared to the 4 replicas of the peptide bonded dimers used in the original ORNL examination. Both forms of the molecule (the monomer and the peptide bonded dimer) are zwitterionic at the examined pH levels, with a deprotonated carboxylic acid functional group and a positively charged primary amine functional group. In total, all of our models contain 84 SOM molecules, 4 duplicates of each SOM molecule, and 8 duplicates of the peptide glycylcysteine.

Particular care has been taken to set the correct protonation of the numerous functional groups contained within the ORNL SOM model. Figure S2 presents the relative concentration of variably charged functional groups contained within the SOM model, where the pKa values for each functional group has been estimated using the MarvinSketch software package from ChemAxon (https://www.chemaxon.com). Between the pH range of 5.5 to 7.5, which encompasses the pertinent environmental variables examined in this study, the sole change to the SOM model is within a phosphate functional group belonging to the *propan-2-yl phosphate* molecule, with a pKa of 6.81, with the functional group being in a $[H_2PO_4{}^-]$ form at low pH values, and in a $[HPO_4{}^{2-}]$ form at neutral and higher pH values.

Setup of Mineral-Associated Organic Matter. Four separate systems were examined in the present study. The SOM model described above was modeled interacting with the iron-oxyhydroxide mineral goethite, setup at a pH of 5.5; as well as the 2:1 TOT phyllosilicate mineral montmorillonite, setup at a pH of 7.5. These two pH levels approximate key mineral-SOM interactions hypothesized as per big-data correlations observed by Rasmussen *et al.*, (2). Furthermore, at the two selected pH levels, all modeled SOM molecules are expected to be present in one dominant protonation state (exceeding a concentration of 80% in all instances). Consequently, all organics were set to their dominant protonation state for the respective system pH. In all models, the excess charge of the system (that of the SOM and, if present, of the negatively charged montmorillonite clay) has been charge balanced by either Na^+ or Ca^{2+} ions, creating four unique models, namely: SOM-Na-goethite; SOM-Ca-goethite; SOM-Na-montmorillonite; SOM-Ca-montmorillonite.

The clay mineral surface of montmorillonite was produced by replicating the unit cell structure of pyrophyllite (Lee & Guggenheim(3)) $8 \times 5 \times 1$ times to create a slab of approximately $4.13 \times 4.48 \times 0.93$ nm^3. Subsequently, randomly selected octahedral aluminum atoms were replaced with magnesium atoms to reproduce a stoichiometry of $(Si_8)(Al_{3.2}Mg_{0.8})O_{20}(OH)_4$, ensuring that magnesiums atoms were placed as to avoid adjacent octahedral isomorphic substitutions.

We have examined the standard charge neutral 1×1 termination of the (010) surface of the goethite as it is one of the most stable and well-examined low-index cleavage structures of this iron-oxyhydroxide(4). We note that the *Pbnm* (010) surface of goethite examined in this study is frequently presented as the (100) surface in the *Pnma* space group in other works(5). Once correctly orientated and cleaved, the unit cell structure of goethite was replicated as to create a system with similar xy-surface area as the clay mineral described above. That is, the unit cell was replicated $14 \times 10 \times 4$ times to create a surface of dimensions approximately $4.23 \times 4.60 \times 0.995$ nm^3. We note that the basal surface of goethite is expected to be terminated with structural -OH_2 ligand molecules at pH 5.5(6). Our model recreates this cleavage structure, whereby the atoms of the -OH_2 ligand are topologically identical to water molecules in the MD simulations.

Four molecules of each SOM constituent (and eight of the peptide glycylcysteine, as per the previous discussion) were placed above the mineral surface within approximately 2 nm of the surface. Three thousand water molecules were then added above the mineral surface surrounding the SOM molecules, and extending up to approximately 6 nm from the mineral surface. Additionally, three hundred and twenty water molecules were placed below each mineral surface. Finally, cations were placed as to charge balance the system. The SOM phase has a net negative charge that is charge balanced with the associated ions of the mineral surface (*i.e.*, all cations in the system are of the same type) and were initially placed within the aqueous SOM phase. For minerals with a net negative charge (*c.f.* the clay minerals), the charge of the mineral was balanced equally both above and below the mineral. Goethite is neutral in this model and consequently no additional charge balancing cations were added beyond those required to balance the charge of the SOM. A vapor phase was placed above the aqueous SOM phase to mimic unsaturated conditions and to minimize interactions between the periodic images of the simulated systems. All initial structures were generated using the software package PACKMOL(7). The overall dimensions of all systems are approximately equivalent, measuring approximately $4.2 \times 4.5 \times 10$ nm^3. An example of the initial starting configuration for the SOM-Na-montmorillonite system is presented in Figure S3.

The Replica-Exchange Molecular Dynamics Procedure. Molecular dynamics (MD) simulations were performed using GROMACS 2022.4(8, 9) on the DOE's Deception HPC at the Pacific Northwest National Laboratory, utilizing the the CHARMM3.6 derived CGenFF force field for SOM molecules(10, 11), the ClayFF force field for minerals and aqueous cations(12), and the SPC/E water model(13).

Unless otherwise stated, all simulations were carried out in the NVT ensemble with a time step of 1 fs using periodic boundary conditions, with electrostatic and Lennard-Jones interactions truncated at a cutoff distance of 1 nm, and with long-range interactions extrapolated using the particle-mesh Ewald summation method. Interaction potentials were shifted at the cutoff to avoid any non-linear jumps in forces between atoms. Off-diagonal Lennard-Jones interactions were calculated using Lorentz-Berthelot mixing rules.

All models were initialized subject to an energy minimization at 0 K to reduce any unphysical overlap between adjacent atoms that may cause further MD simulations to fail. Convergence was satisfied once the maximum force between any two atoms in the system decreased beneath a limit of 1000.0 kJ/mol/nm. A short 100 ps molecular dynamics simulation was then performed to bring the system up to 300 K using a velocity-rescale thermostat with a temperature coupling constant of 0.1 ps.

Each system was subsequently replicated 8 times to perform a 100 ns replica-exchange equilibration. The REMD simulations were calculated with exchanges between adjacent temperatures attempted every 1 ps (1000 time steps). The temperature of each replicate varied in 5 K increments between 300 and 335 K, and the simulations were performed using a velocity-rescale thermostat with a temperature coupling constant of 0.1 ps. On average, we observed an approximately 2% successful swap rate between the lowest temperature configurations (300 and 305 K), increasing to an approximately 3.5% successful swap rate between the highest temperature configurations (330 and 335 K).

Following the REMD simulations, each temperature replicate was extended in a production simulation using the traditional MD simulation technique for 200 ns. Atomic positions, velocities, and forces were saved every 5 ps. Data analyses were applied over all temperature replicates and time steps from these production trajectories.

Analyses. To analyze the resulting configurations, we developed a novel clustering algorithm capable of describing whether an SOM molecule is aggregated on a mineral surface and, if so, whether it is directly sorbed, bridged through an intermediate cation, or sorbed through auxiliary intermediate SOM molecules. The method is based off calculating the minimum intramolecular distances between all pairs of molecules in the system (including SOM molecules, charge-balancing cations, and the mineral surface) and comparing these values against a predefined distance cutoff.

First, we quantified the ions that are directly sorbed to the mineral surface within the Stern layer. This information was subsequently used to calculate SOM molecules sorbed to the mineral through the formation of cation bridges. For Na^+ ions, a cutoff distance of 5Å between ion and mineral was set; for calcium ions, a cutoff distance of 4.5Å was set. These cutoff values correspond to the first density minimum for sodium and calcium ions adjacent to a montmorillonite basal surface in a bulk water system (data not presented). Additionally, the cutoff values accurately quantify the sorption of each respective ion to the goethite surface.

Second, all atoms of the SOM phase were examined. To calculate the sorption of SOM to the mineral, the minimum atomic distances between each SOM molecule and the mineral surface were calculated. If any of these distances were lower than a set cutoff distance (see later discussion), then the SOM molecules were tagged as *directly sorbed*. Subsequently, we reproduce the same minimum atomic distance calculation between all SOM phase molecules and the ions previously tagged as sorbed to the mineral in the Stern Layer. This allows us to calculate which SOM phase molecules were *cation bridged* to the mineral surface.

Third, we examined and quantified the clustering of SOM in the bulk phase. The minimum distance between atoms in every pairwise intramolecular SOM and cation combination were calculated and compared against a minimum cutoff distance. If the minimum distance between any two atoms within a combinatorial pair are lower than the predefined cutoff distance, then these two molecules were tagged as *paired*. Once the calculation had looped through all combinations of cations and SOM phase molecules, the lists of *paired* molecules were compared and combined to calculate the clusters of SOM throughout the system. This was achieved using network analysis python package *networkx*(14). In all cases, the predefined cutoff distance was set to 4.84Å, the approximate diameter of a water molecule in a simulation of bulk SPC/E at 298K. Finally, we quantified the hydrophobically partitioned sorption of SOM to a mineral surface. This was categorized as the SOM phase molecules that were a component of a cluster sorbed to the mineral, but that had not been tagged as either directly sorbed or cation bridged to the surface.

Details pertaining to the sorption and clustering of SOM were correlated to the chemical features of each SOM molecule calculated using the RDKit cheminformatics toolkit. The use of RDKit permitted us to automatically correlate the partitioning of SOM against several metrics such as molecular mass, a molecule's elemental composition, and a molecule's functional group content. We observe monotonic relationships between the SOM's probability of clustering and numerous chemical features. Consequently, we quantified the degrees of correlation using Spearman correlation coefficients.

To quantify the existence of aromatic moieties in our models we calculated the degree of unsaturation, also known as the double-bond equivalence (DBE), for each molecule listed in Table S1. The calculation follows from the description given by Koch & Dittmar(15), and is given by the relation:

$$\mathrm{DBE} = 1 + \frac{1}{2}\left(2\mathrm{C} - \mathrm{H} + \mathrm{N} + \mathrm{P}\right) \qquad [1]$$

In-house tools were utilized to calculate the solvent accessible surface area (SASA) of each molecule, discretized into polar and apolar SASA's. This was achieved using the *rdFreeSASA* module of RDKit linked to the software package FreeSASA(16). A molecule's SASA was estimated using the algorithm of Lee & Richards(17). The polar SASA of a molecule was defined as that corresponding to oxygen or nitrogen atoms and the apolar surface area is that corresponding to other atoms. Hydrogen atoms were excluded in this calculation.

The octanol-water partition coefficient of each SOM molecule was predicted using the XLOGP3 software package(18).

Finally, we developed a method utilizing Voronoi tessellation to discretize the surface area coverage of each mineral. In so doing, we quantify how much of a mineral's surface area is interfaced with water, with ions, and with SOM molecules. First, we identified the first four atomic layers of the aqueous phase (including SOM, cations, and water) above each mineral surface using the PyTIM algorithm(19). An example of this process is presented in Figure S4. Second, we perform a Voronoi tessellation on the {x, y} coordinates of all atoms within each of the four interfacial layers. An example is presented in Figure S5. Finally, we calculated the surface area in each of the four interfacial layers corresponding to water molecules, SOM molecules, and cations.

Additional Results

Figure S6 presents the probability of SOM molecules partitioning into clusters as a function of cluster size, averaged over all temperature replicates. Notably, we observe a large primary cluster of SOM forming in each system. The average size of this cluster is larger in the systems containing calcium charge balancing cations. Additionally, we note that our clustering algorithm is capable of calculating the probability of SOM molecules existing as monomers.

Figure S7 presents the surface area coverage in the first four interfacial atomic layers above the mineral surfaces of montmorillonite and goethite. Notably, we observe that water retains significant access to the mineral surface in all of the examined systems.

Figure S8 presents the matrix of the Spearman correlation coefficients calculated between key molecular properties of the SOM phase and the probability of SOM (a) partitioning into the largest primary cluster in the system - columns with the **cluster** suffix, or (b) being a component of the contact zone - columns with the **bridging** suffix. The row indices of Figure S8 prefixed with **fr** indicate the presence a functional group in an SOM molecule. These prefixes correspond to the functional groups listed in Table S2.

Table S2. Keywords for quantifying functional groups as per Figure S8.

Keyword	Functional Group
fr_Al_COO	Aliphatic carboxylic acids
fr_Al_OH	Aliphatic hydroxyl groups
fr_Ar_COO	Aromatic carboxylic acide
fr_Ar_N	Aromatic nitrogens
fr_Ar_OH	Aromatic hydroxyl groups
fr_COO	Carboxylic acids
fr_COO2	Carboxylic acids
fr_C_O	Carbonyl O
fr_C_O_noCOO	Carbonyl O, excluding COOH
fr_NH0	Tertiary amines
fr_NH1	Secondary amines
fr_NH2	Primary amines
fr_SH	Thiol groups
fr_amide	Amides
fr_aniline	Anilines
fr_benzene	Benzene rings
fr_bicyclic	Bicyclic
fr_ester	Esters
fr_ether	Ether oxygens (including phenoxy)
fr_ketone	Ketones
fr_ketone_Topliss	ketones excluding diaryl, a,b-unsat. dienones, heteroatom on Calpha
fr_methoxy	Methoxy groups -OCH3
fr_para_hydroxylation	Para-hydroxylation sites
fr_phenol	Phenols
fr_phenol_noOrthoHbond	Phenolic OH excluding ortho intramolecular Hbond substituents
fr_phos_acid	Phosphoric acid groups
fr_phos_ester	Phosphoric ester groups
fr_pyridine	Pyridine rings

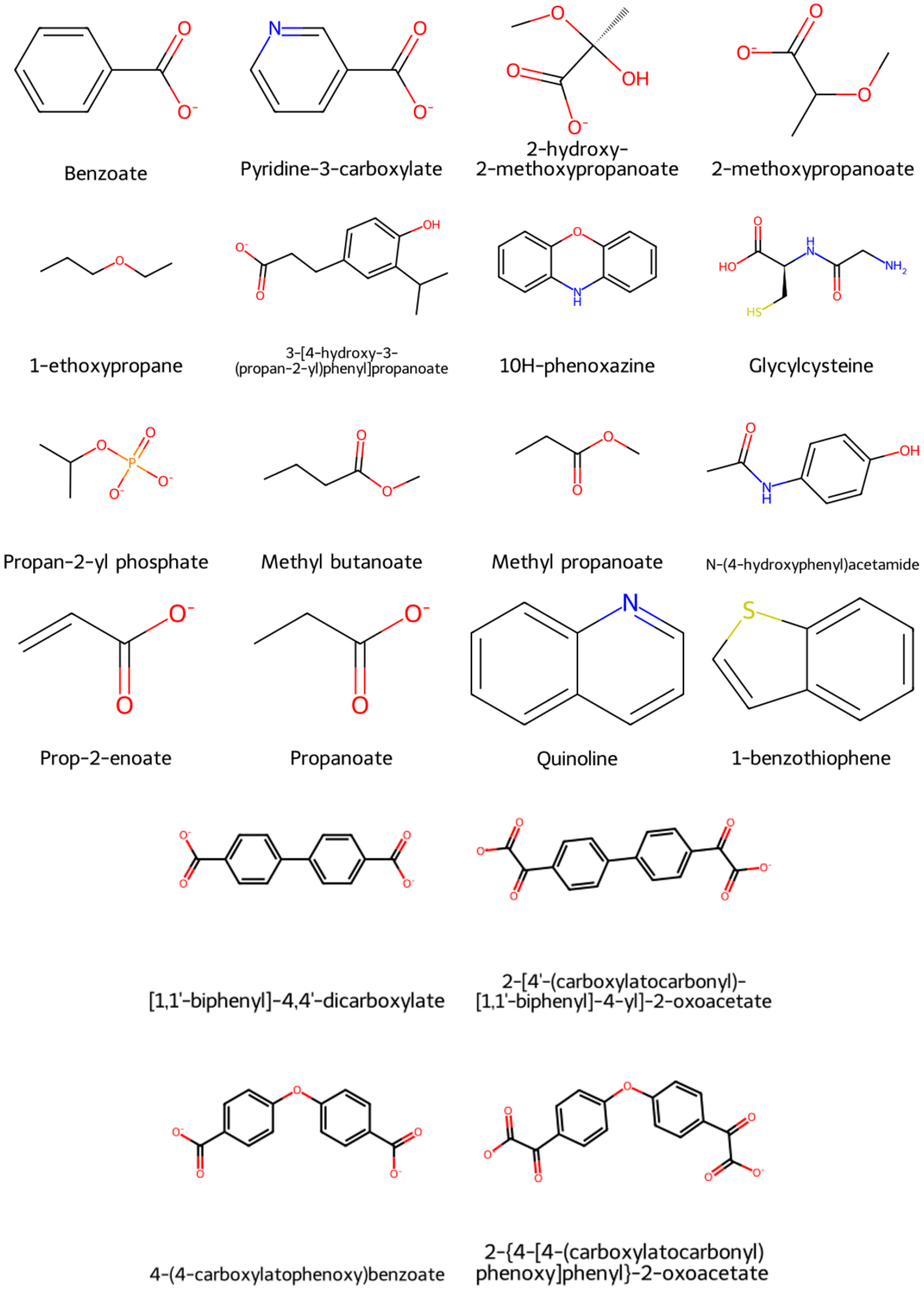

Fig. S1. The structure of every SOM molecule used in the present study.

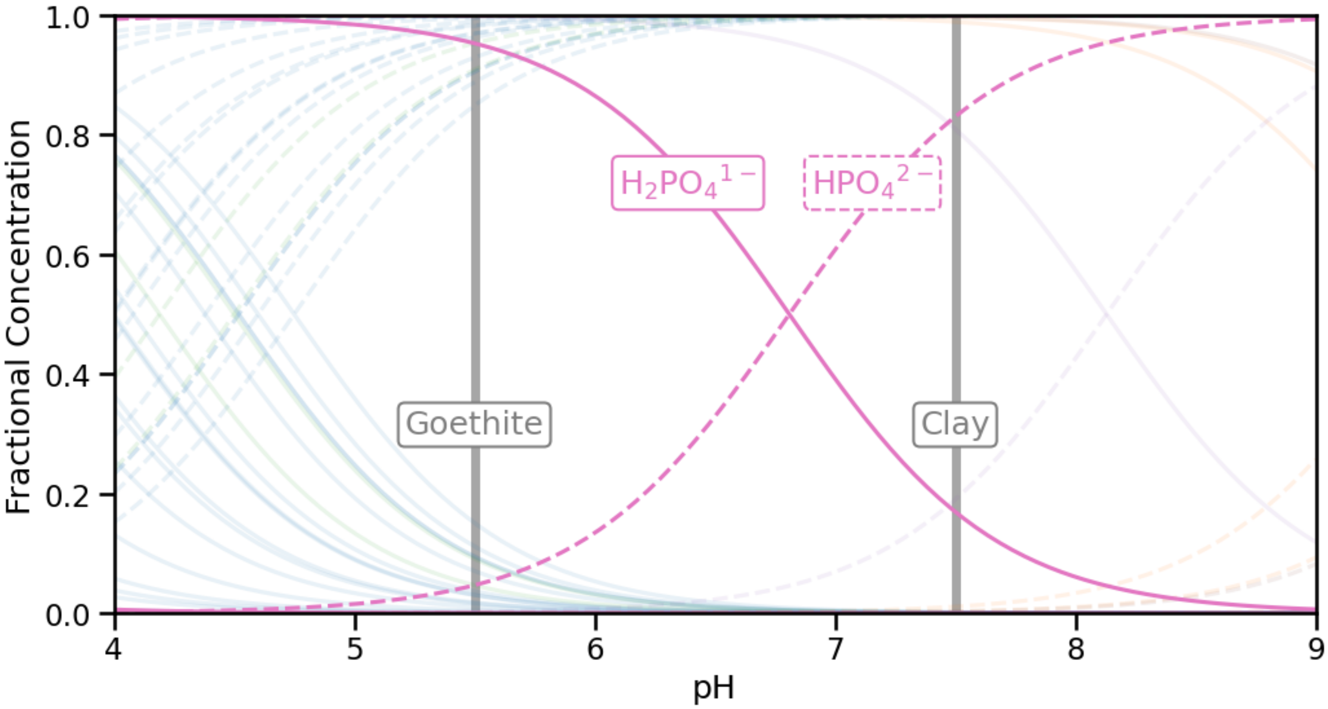

Fig. S2. Henderson-Hasselbalch plots for the ionizable functional groups in the ORNL SOM model. Highlighted in pink is the phosphate functional group of propan-2-yl phosphate (pKa = 6.81). The simulations have been setup to approximate environmental conditions, *c.f.* a pH of approximately 5.5 for goethite systems and 7.5 for clay systems. In the models, all organics are set to their prominent protonation state for the respective system pH.

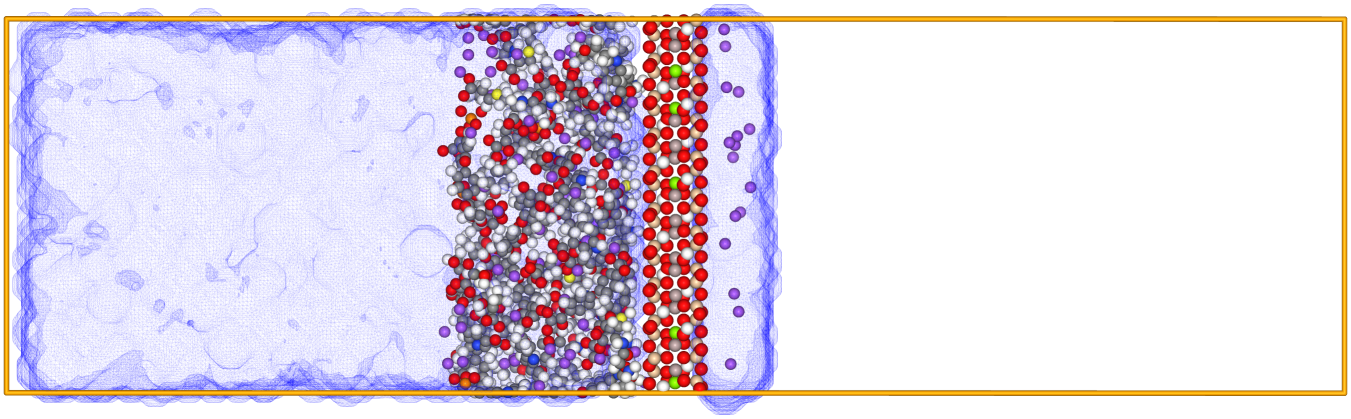

Fig. S3. The initial starting structure of the SOM-Na-montmorillonite system. Spheres represent SOM atoms (C, O, H, N, S, and P in grey, red, white, blue, yellow, and orange respectively); the clay mineral atoms (O, H, Si, Al, Mg in red, white, tan, taupe, and green respectively); and charge-compensating Na^{+} ions (purple); water molecules are not shown, however the extent of the water phase is presented in the transparent blue region.

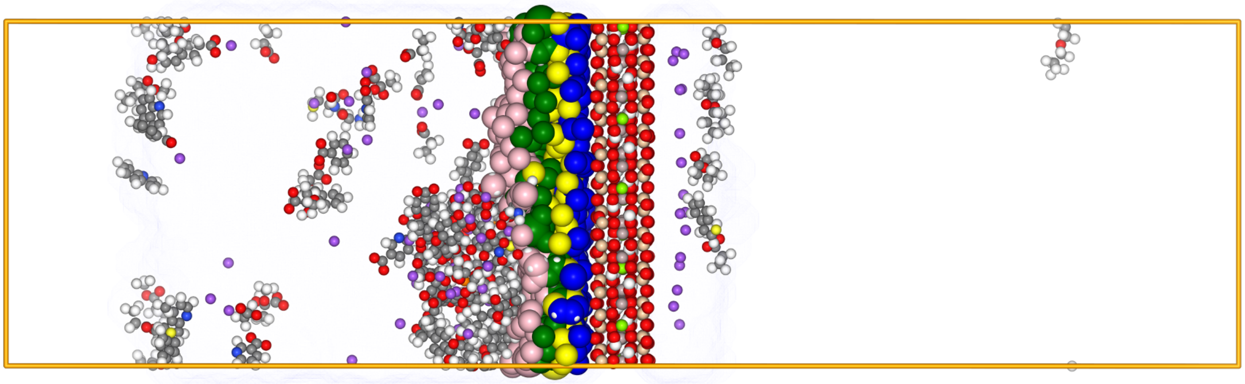

Fig. S4. An example of quantifying the interfacial atoms above the mineral surface of Na-montmorillonite using the PyTIM algorithm. The first, second, third, and fourth interfacial layers are tagged as large spheres of blue, yellow, green, and pink respectively. All other atoms are colored as per Figure S3.

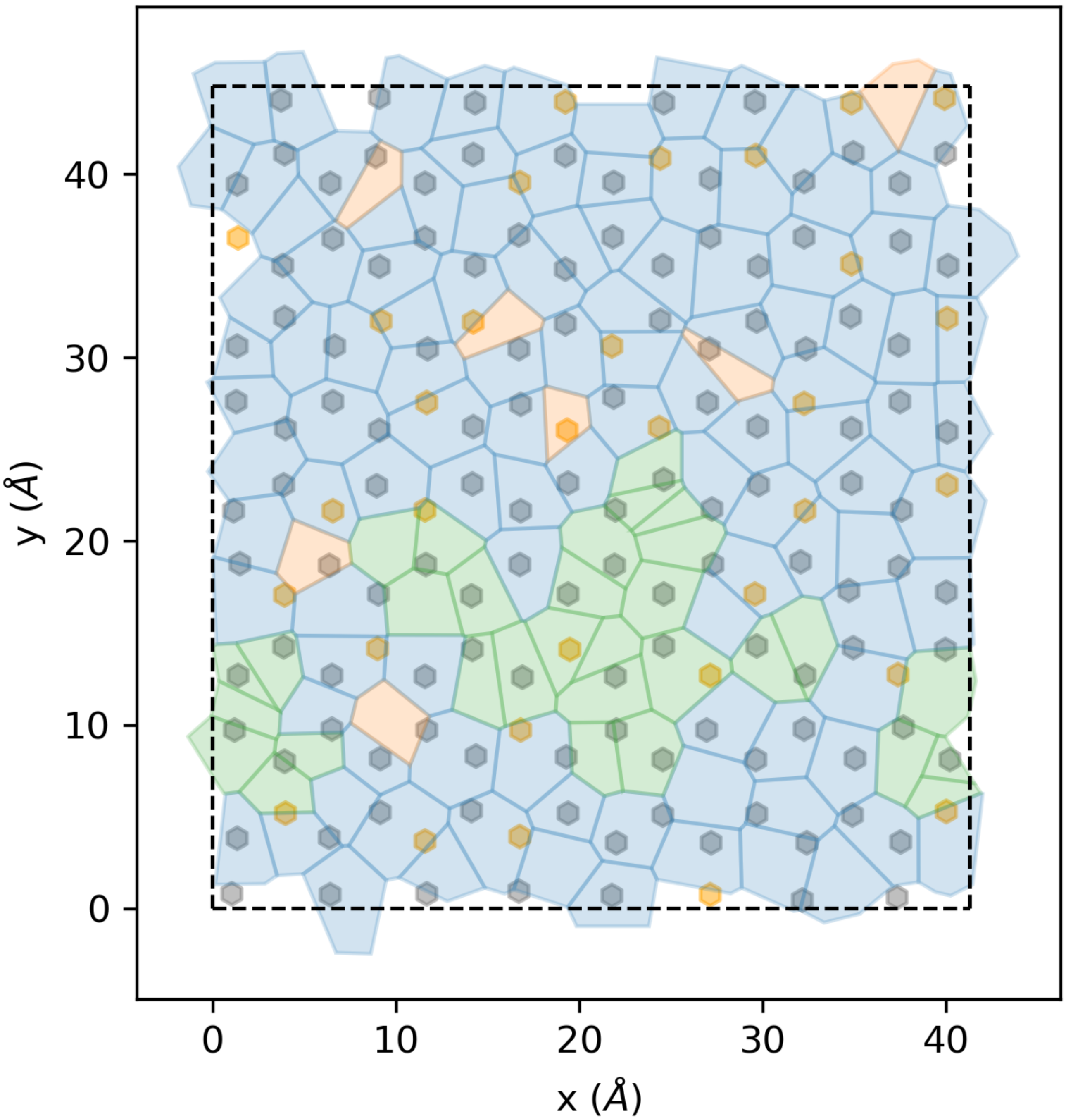

Fig. S5. An example of the Voronoi surface area discretization and calculation for the first atomic layer adjacent to Na-montmorillonite. Blue regions represent the area covered by water, orange by sodium ions, and green by SOM atoms. The underlying octahedral metal atoms of the montmorillonite mineral are also presented as hexagonally-arranged dots, with aluminum atoms in grey, and isomorphically substituted magnesium atoms (*i.e.*, sites of negative structural charge) in orange. The dotted black line shows the periodic boundary of the simulation.

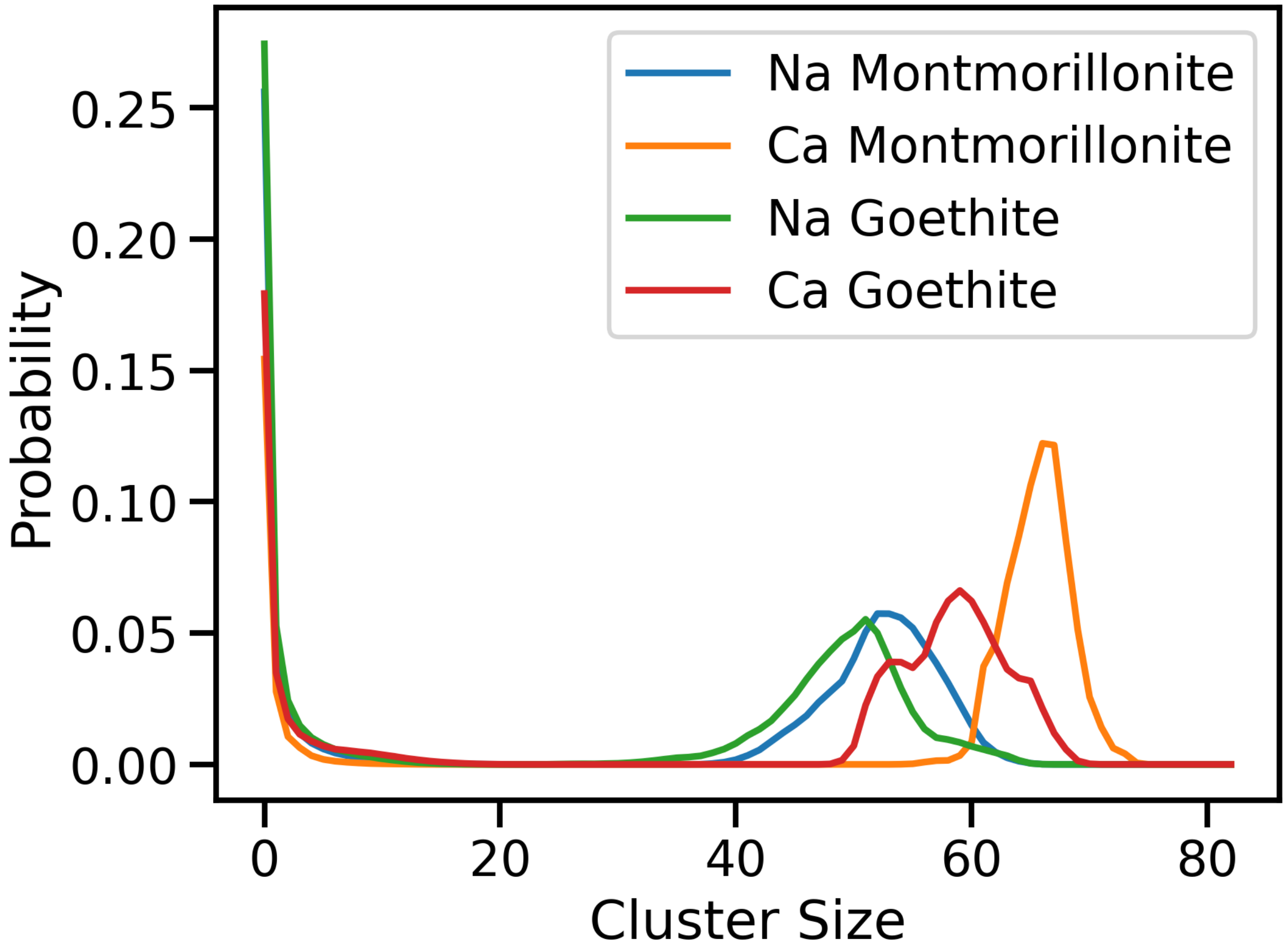

Fig. S6. The cluster-size distribution probability calculated over all SOM molecules in a system. Data has been averaged over all temperature ranges.

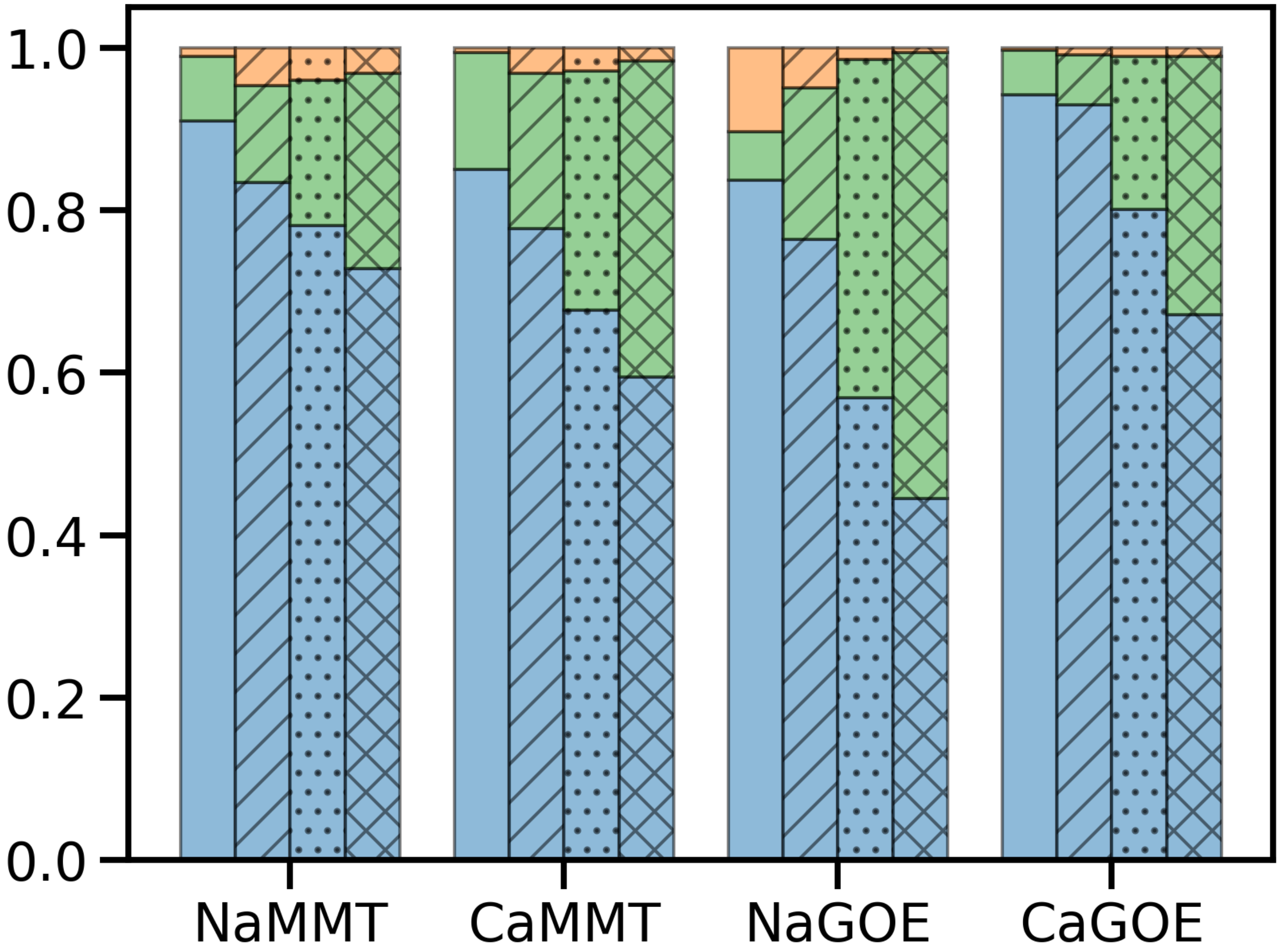

Fig. S7. The percentage surface area coverage of the first four atomic interfacial areas about the surfaces of montmorillonite and goethite. Data has been averaged over all temperature ranges. Non-hashed, forward-slashed hashed, dotted hashed, and cross-slashed hashed columns represent the first, second, third, and fourth atomic layer respectively. Blue, green, and orange regions represent the surface area percentage corresponding to water, SOM molecules, and cations respectively.

	naMMT_cluster	caMMT_cluster	naGOE_cluster	caGOE_cluster	naMMT_bridging	caMMT_bridging	naGOE_bridging	caGOE_bridging
**Charge**	-0.582144	-0.748714	-0.373543	-0.515613	0.232858	-0.345128	-0.670852	-0.541256
**Mass**	0.768831	0.617084	0.836364	0.718182	0.435065	0.327273	0.490909	0.350649
**C**	0.621991	0.480875	0.785501	0.625915	0.429704	0.333560	0.433628	0.315901
**H**	0.102256	-0.028377	0.253990	0.138540	0.048159	-0.090381	-0.078506	-0.170206
**O**	0.484511	0.644450	0.425279	0.560383	-0.250908	0.309475	0.618285	0.466542
**N**	0.178538	0.026599	0.243116	0.132954	0.607790	0.281103	-0.148149	0.041786
**S**	0.000000	-0.107195	0.053580	0.026790	0.509010	0.133950	-0.160740	0.026790
**P**	0.000000	-0.107195	-0.241110	-0.214320	-0.160740	-0.428640	-0.187530	-0.294690
**Aromatic**	0.598345	0.409527	0.740058	0.566853	0.551107	0.299172	0.393648	0.299172
**PolarSASA**	0.558442	0.689185	0.596104	0.706494	-0.044156	0.445455	0.683117	0.583117
**ApolarSASA**	0.612987	0.434557	0.706494	0.537662	0.397403	0.193506	0.320779	0.176623
**XLogP3**	0.480676	0.489929	0.617733	0.550828	0.100032	0.316986	0.565768	0.464437
**SASA**	0.729870	0.569016	0.848052	0.711688	0.431169	0.309091	0.490909	0.342857
**Apolar%**	-0.044156	-0.277363	0.031169	-0.177922	0.284416	-0.262338	-0.311688	-0.349351
**Polar%**	0.044156	0.277363	-0.031169	0.177922	-0.284416	0.262338	0.311688	0.349351
**H/C**	-0.597658	-0.579617	-0.701825	-0.646487	-0.335288	-0.504559	-0.595054	-0.558596
**O/C**	-0.173602	0.085203	-0.330950	-0.094278	-0.514305	-0.002601	0.094278	0.094929
**DBE**	0.681025	0.605552	0.795181	0.683634	0.478805	0.523163	0.571435	0.531643
**DBE/C**	0.527824	0.521160	0.584447	0.570128	0.380736	0.587050	0.561017	0.625449
**AI**	0.411357	0.152087	0.370550	0.276432	0.546282	0.127685	0.108598	0.151379
**fr_Al_COO**	0.099432	0.284394	0.210649	0.293877	-0.042719	0.283565	0.259996	0.234218
**fr_Al_OH**	-0.221565	-0.147758	-0.221565	-0.221565	-0.258492	-0.036927	-0.073855	-0.110782
**fr_Ar_COO**	0.345201	0.381837	0.385032	0.399258	-0.022761	0.242779	0.482713	0.460901
**fr_Ar_N**	-0.053580	-0.133993	-0.053580	-0.107160	0.375060	-0.026790	-0.187530	-0.053580
**fr_Ar_OH**	0.026790	0.053597	0.053580	0.080370	0.000000	0.348270	0.214320	0.294690
**fr_COO**	0.463261	0.666417	0.597621	0.678097	-0.083975	0.478657	0.713786	0.650105
**fr_COO2**	0.463261	0.666417	0.597621	0.678097	-0.083975	0.478657	0.713786	0.650105
**fr_C_O**	0.449654	0.642116	0.619602	0.714490	-0.001416	0.608272	0.720863	0.713781
**fr_C_O_noCOO**	0.434872	0.640537	0.594591	0.684614	-0.108899	0.546675	0.737612	0.686066
**fr_NH0**	-0.053580	-0.133993	-0.053580	-0.107160	0.375060	-0.026790	-0.187530	-0.053580
**fr_NH1**	0.022473	-0.067442	0.044947	0.000000	0.337100	0.359573	-0.067420	0.157313
**fr_NH2**	0.073855	0.073879	0.147710	0.184637	0.369274	0.332347	0.036927	0.221565
**fr_SH**	0.073855	0.073879	0.147710	0.184637	0.369274	0.332347	0.036927	0.221565
**fr_amide**	-0.053580	-0.026799	-0.026790	0.053580	0.187530	0.428640	0.080370	0.348270
**fr_aniline**	-0.017838	-0.132551	-0.043320	-0.132508	0.147798	0.178377	-0.117219	0.010193
**fr_benzene**	0.600901	0.524593	0.678836	0.617653	0.189375	0.419538	0.579778	0.475622
**fr_bicyclic**	0.033127	-0.209023	0.033127	-0.157991	0.453586	-0.101929	-0.346560	-0.295595
**fr_ester**	-0.133950	-0.187591	-0.080370	-0.133950	0.026790	-0.160740	-0.214320	-0.267900
**fr_ether**	-0.064775	0.032398	-0.080969	-0.113356	-0.064775	-0.113356	-0.161938	-0.307681
**fr_ketone**	0.401850	0.442178	0.455430	0.509010	0.053580	0.241110	0.428640	0.267900
**fr_ketone_Topliss**	0.401850	0.442178	0.455430	0.509010	0.053580	0.241110	0.428640	0.267900
**fr_methoxy**	-0.240321	-0.140233	-0.260347	-0.240321	-0.140187	0.020027	-0.220294	-0.240321
**fr_para_hydroxylation**	0.033127	-0.209023	0.033127	-0.157991	0.453586	-0.101929	-0.346560	-0.295595
**fr_phenol**	0.026790	0.053597	0.053580	0.080370	0.000000	0.348270	0.214320	0.294690
**fr_phenol_noOrthoHbond**	0.026790	0.053597	0.053580	0.080370	0.000000	0.348270	0.214320	0.294690
**fr_phos_acid**	0.369274	0.221637	-0.369274	-0.369274	0.147710	-0.221565	-0.369274	-0.369274
**fr_phos_ester**	0.369274	0.221637	-0.369274	-0.369274	0.147710	-0.221565	-0.369274	-0.369274
**fr_pyridine**	-0.053580	-0.133993	-0.053580	-0.107160	0.375060	-0.026790	-0.187530	-0.053580

Fig. S8. Spearman correlation coefficients between multiple cheminformatic measures of each SOM molecule; whether the SOM molecule is in the primary cluster; and whether the SOM molecule is bridging to the mineral surface. Cells shaded in blue present a negative correlation, cells shaded in red present a positive correlation. Row indices prefixed with **fr** indicate the presence of the pertinent functional group in an SOM molecule.

References

1. D Devarajan, et al., Molecular Dynamics Simulation of the Structures, Dynamics, and Aggregation of Dissolved Organic Matter. *Environ. Sci. & Technol.* **54**, 13527–13537 (2020).
2. C Rasmussen, et al., Beyond clay: Towards an improved set of variables for predicting soil organic matter content. *Biogeochemistry* **137**, 297–306 (2018).
3. JH Lee, S Guggenheim, Single crystal X-ray refinement of pyrophyllite-1Tc. *Am. Mineral.* **66**, 350–357 (1981).
4. JD Kubicki, KW Paul, DL Sparks, Periodic density functional theory calculations of bulk and the (010) surface of goethite. *Geochem. Transactions* **9**, 4 (2008).
5. JD Kubicki, D Tunega, S Kraemer, A density functional theory investigation of oxalate and Fe(II) adsorption onto the (010) goethite surface with implications for ligand- and reduction-promoted dissolution. *Chem. Geol.* **464**, 14–22 (2017).
6. Y Zhang, X Liu, J Cheng, X Lu, Interfacial structures and acidity constants of goethite from first-principles Molecular Dynamics simulations. *Am. Mineral.* **106**, 1736–1743 (2021).
7. L Martínez, R Andrade, EG Birgin, JM Martínez, PACKMOL: A package for building initial configurations for molecular dynamics simulations. *J. Comput. Chem.* **30**, 2157–2164 (2009).
8. D Van Der Spoel, et al., GROMACS: Fast, flexible, and free. *J. Comput. Chem.* **26**, 1701–1718 (2005).
9. MJ Abraham, et al., GROMACS: High performance molecular simulations through multi-level parallelism from laptops to supercomputers. *SoftwareX* **1–2**, 19–25 (2015).
10. K Vanommeslaeghe, ADJr MacKerell, Automation of the CHARMM General Force Field (CGenFF) I: Bond Perception and Atom Typing. *J. Chem. Inf. Model.* **52**, 3144–3154 (2012).
11. K Vanommeslaeghe, EP Raman, ADJr MacKerell, Automation of the CHARMM General Force Field (CGenFF) II: Assignment of Bonded Parameters and Partial Atomic Charges. *J. Chem. Inf. Model.* **52**, 3155–3168 (2012).
12. RT Cygan, JJ Liang, AG Kalinichev, Molecular Models of Hydroxide, Oxyhydroxide, and Clay Phases and the Development of a General Force Field. *The J. Phys. Chem. B* **108**, 1255–1266 (2004).
13. HJC Berendsen, JR Grigera, TP Straatsma, The missing term in effective pair potentials. *The J. Phys. Chem.* **91**, 6269–6271 (1987).
14. A Hagberg, PJ Swart, DA Schult, Exploring network structure, dynamics, and function using NetworkX, (Los Alamos National Laboratory (LANL), Los Alamos, NM (United States)), Technical Report LA-UR-08-05495; LA-UR-08-5495 (2008).
15. BP Koch, T Dittmar, From mass to structure: An aromaticity index for high-resolution mass data of natural organic matter. *Rapid Commun. Mass Spectrom.* **20**, 926–932 (2006).
16. S Mitternacht, FreeSASA: An open source C library for solvent accessible surface area calculations (2016).
17. B Lee, FM Richards, The interpretation of protein structures: Estimation of static accessibility. *J. Mol. Biol.* **55**, 379–IN4 (1971).
18. T Cheng, et al., Computation of Octanol-Water Partition Coefficients by Guiding an Additive Model with Knowledge. *J. Chem. Inf. Model.* **47**, 2140–2148 (2007).
19. M Sega, G Hantal, B Fábián, P Jedlovszky, Pytim: A python package for the interfacial analysis of molecular simulations. *J. Comput. Chem.* **39**, 2118–2125 (2018).
